# Perception of Arm Position in Three-Dimensional Space

**DOI:** 10.3389/fnhum.2018.00331

**Published:** 2018-08-21

**Authors:** Joshua Klein, Bryan Whitsell, Panagiotis K. Artemiadis, Christopher A. Buneo

**Affiliations:** ^1^Interdisciplinary Graduate Program in Neuroscience, Arizona State University, Tempe, AZ, United States; ^2^Alliance for Person-Centered Accessible Technologies, Arizona State University, Tempe, AZ, United States; ^3^School for Engineering of Matter, Transport and Energy, Arizona State University, Tempe, AZ, United States; ^4^School of Biological and Health Systems Engineering, Arizona State University, Tempe, AZ, United States

**Keywords:** proprioception, robotics, rehabilitation, position sense, psychophysics

## Abstract

Proprioception refers to the senses of body position, movement, force and effort. Previous studies have demonstrated workspace and direction-dependent differences in arm proprioceptive sensitivity within the horizontal plane. In addition, studies of reaching in the vertical plane have shown that proprioception plays a key role in anticipating arm configuration dependent effects of gravity. This suggests that proprioceptive sensitivity could vary with the direction of arm displacement relative to the gravitational vector, as well as with arm configuration. To test these hypotheses, and to characterize proprioception more generally, we assessed the direction-dependence and arm postural-dependence of proprioceptive sensitivity in 3D space using a novel robotic paradigm. A subject’s right arm was coupled to a 7-df robot through a trough that stabilized the wrist and forearm, allowing for changes in configuration largely at the elbow and shoulder. Sensitivity was evaluated using a “same-different” task, where the subject’s hand was moved 1–4 cm away from an initial “test” position to a 2nd “judgment” position. The proportion of trials where subjects responded “different” when the positions were different (“hit rate”), and where they responded “different” when the positions were the same, (“false alarm rate”), were used to calculate d’, a measure of sensitivity derived from signal detection theory (SDT). Initially, a single initial arm posture was used and displacements were performed in six directions: upward, downward, forward, backward, leftward and rightward of the test position. In a follow-up experiment, data were obtained for four directions and two initial arm postures. As expected, sensitivity (d’) increased monotonically with distance for all six directions. Sensitivity also varied between directions, particularly at position differences of 2 and 3 cm. Overall, sensitivity reached near maximal values in this task at 2 cm for the leftward/rightward directions, 3 cm for upward/forward and 4 cm for the downward/backward directions. In addition, when data were grouped together for opposing directions, sensitivity showed a dependence upon arm posture. These data suggest arm proprioceptive sensitivity is both anisotropic in 3D space and configuration-dependent, which has important implications for sensorimotor control of the arm and human-robot interactions.

## Introduction

Proprioception refers to the senses of body position (“position sense”), movement (“kinethesis”) and force/effort/heaviness (Proske and Gandevia, 2012). Loss or impairment of proprioception is a natural sequela of a host of conditions affecting both the central nervous system (CNS) and peripheral nervous system (PNS) including stroke, traumatic brain injury, Parkinson’s disease, diabetes and even certain orthopedic injuries. Loss of this “sixth sense” impairs perception of the relative configurations of body parts in space (“body schema”) and dramatically affects the planning and control of limb and body movement (Ghez et al., 1995; Gordon et al., 1995). This in turn has profoundly negative effects on the performance of essential activities of daily living, leading to reduced quality of life.

Despite its importance for normal sensorimotor functioning, proprioception remains enigmatic and its assessment in the clinic remains relatively crude. Robotic technologies have recently been employed in an attempt to improve the fidelity of clinical assessments of proprioception. For example, Dukelow et al. (2012) have developed a version of the classic position matching paradigm that employs the use of planar robotic exoskeletal arms (Dukelow et al., 2010). In this paradigm, one exoskeletal arm passively moves the test arm into a test position and the subject then attempts to actively match this position with the other arm. Analysis focuses on quantifying differences between the positions generated by the passively and actively moved arms. In experiments comparing the proprioceptive abilities of stroke survivors with age-matched controls, this method was found to have good interrater reliability and revealed that approximately one half of examined patients exhibited some degree of proprioceptive (position sensing) impairment (Dukelow et al., 2010).

Other investigators have combined the use a planar robotic manipulandum with sensory psychophysical techniques to assess proprioception. For example, one recent study compared proprioceptive function between a group of neurologically intact human subjects and a group of stroke survivors (Simo et al., 2014). Proprioception was probed using both an arm movement detection and a hand force detection task. Subject performance was quantified using two parameters: detection threshold, which is the minimum magnitude of displacement or forces that can be reliably detected, and choice uncertainty, the variability in responses about the detection threshold. These measures were able to distinguish between subjects with and without proprioceptive deficits and were found to be relatively reliable in repeated tests separated by a period of 1 week.

Robotic devices have also been used to aid in understanding the proprioceptive abilities of neurologically intact subjects (Dukelow et al., 2010, 2012; Fuentes and Bastian, 2010; Wilson et al., 2010; Cressman and Henriques, 2011; Simo et al., 2014). Most of these studies have focused on proprioceptive abilities within a single horizontal plane and have demonstrated (among other findings) that proprioceptive sensitivity depends on both the position of the arm and the direction of arm displacement within the 2D workspace. Although wrist proprioception has recently been characterized in 3D (Marini et al., 2016), similar tests for the proximal arm have yet to be conducted. However, recent studies have shown that arm kinematics vary for movements performed along different directions in the vertical plane (i.e., with and against the direction of the gravity vector) in a manner that is consistent with an optimization of both inertial *and* gravitational forces (Papaxanthis et al., 2003; Gentili et al., 2007; Le Seac’h and Mcintyre, 2007; Berret et al., 2008). Moreover, other work suggests that anticipating such gravitational effects on the arm depends strongly on input from the proprioceptive system (Soechting, 1982; Soechting and Ross, 1984; Worringham and Stelmach, 1985; Worringham et al., 1987; Swinnen et al., 1997; Lemay et al., 2004; Proske, 2005; Dalecki and Bock, 2013). This raises the possibility that proprioceptive abilities could also differ for movements performed along different directions in the vertical plane, more specifically as a function of direction with respect to the gravitational vector. By a similar logic, proprioceptive sensitivity could vary with changes in arm configuration.

Although previous work suggests that arm proprioceptive sensitivity *could* vary with direction and configuration in 3D space, a formal test of this hypothesis has yet to be conducted. Here, we used a 7 degree of freedom (df) robotic arm, a 1 alternative forced choice (AFC; “same-different”) psychophysical paradigm and analysis techniques derived from signal detection theory (SDT) to perform such a test. In an initial experiment, sensitivity to differences in arm position was quantified and compared for arm displacements along six directions in 3D space: leftward, rightward, forward, backward, upward and downward with respect to a fixed reference position. In a 2nd experiment, sensitivity was compared for four directions (leftward/rightward, forward/backward) and two initial arm postures (adducted and abducted). Preliminary results of these experiments have previously been reported in abstract form (Klein et al., 2015, 2016, 2017).

## Materials and Methods

### Participants

The experimental protocol was approved by the Arizona State University Institutional Review Board and all subjects gave written informed consent in accordance with the Declaration of Helsinki. Subjects were briefed on the experimental procedures and expectations for interacting with the robot and were aware that their position sense was being tested but were naïve to the specific purpose of the study. In an initial experiment (Experiment 1) examining the effects of displacement direction on proprioceptive sensitivity, 78 subjects (49 female, 29 male) were tested for two of the six displacement directions in a given session. Two subjects (one male, one female) were determined to be outliers in both tested directions based on their median absolute deviation and were removed from further analysis. After outlier removal, the total number of subjects analyzed in each direction was as follows: Upward: 30; Downward: 27; Backward: 19; Forward: 15; Leftward: 18; Rightward: 17. In a follow-up experiment (Experiment 2) examining the additional effects of arm posture, 20 subjects (eight female, 12 male) were tested in two of four directions (Backward, Forward, Leftward, Rightward) in an abducted arm posture. One female subject was determined to be an outlier for both tested directions as was removed from further analysis.

### Apparatus

**Figure 1:**
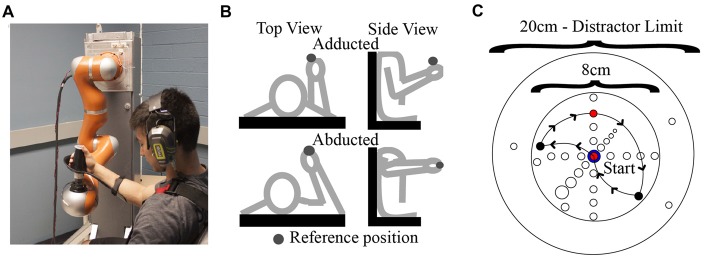
**(A)** Human-robot coupling at the test position. Photograph used with permission of subject. **(B)** Arm postures examined in this study. **(C)** Locations of endpoints and via points (i.e. distractors) with respect to the reference position. An example path taken by the robot on a single trial is also shown (arrows).

A 7-df anthropomorphic robot arm (LWR4+, KUKA Inc.) was used for the robotic assessment (Figure 1A). This robot has a maximum payload of 7 kg, a maximum reach of 1,178 mm (when completely stretched), a maximum joint speed of 110–204°/s (joint dependent) and a repeatability of ± 0.05 mm. The robot can be controlled in zero-impedance, i.e., completely compliant to user’s motion, and is able to measure arm motion and human-robot interaction forces at a frequency of 1 kHz. Subjects interacted with the robot while seated in a chair that could be locked in place and adjusted in height for participant comfort. Human arms were coupled to the robot through an arm trough which was secured to the arm with a Velcro strap and which also stabilized and controlled the forearm and wrist. Excessive motion of the shoulder girdle and trunk were restricted by means of waist and shoulder straps that were attached to the chair. In addition, subjects were given a switch which could be pressed at any time to immediately stop motion of the robot.

In Experiment 1, a single “adducted” arm posture was employed. During an initial calibration procedure, the plane of robot motion was first aligned with a parasagittal plane passing the shoulder joint. The initial test position of the hand-robot coupling was then specified, which was located at approximately 5° azimuth and 0° elevation relative to the estimated (average) center of rotation of the shoulder joint and at a distance from the shoulder that corresponded to ~80% of the subject’s total arm length. In Experiment 2, data were collected for an additional “abducted” arm posture. This was achieved by rotating the upper arm about an axis connecting the shoulder to the hand and suspending the arm with a sling attached by ropes to the ceiling of the testing room. In this way the upper arm and forearm were contained in an approximately horizontal plane as shown in Figure 1B.

### Experimental Procedures

#### Experimental Design

Sensitivity to differences in limb position were evaluated using a fixed “AX” or “same-different” task (also referred to as a 1 AFC same-different task; Macmillan and Creelman, 2005; Micheyl et al., 2008; Kingdom and Prins, 2010; DeCarlo, 2013). This is a discrimination paradigm involving the successive presentation of a pair of stimuli, with half the trials containing stimuli pairs that are the same and half the trials containing pairs that are different. Subjects are required to determine whether the pair presented in a given trial is the “same” or “different” (Kingdom and Prins, 2010). The modifier “fixed” refers to the fact that in this experiment the second stimulus was compared relative to a fixed, standard stimulus (the “test position”), which differs from “roving” designs where both stimuli are varied along a continuum. The same-different task requires the detection of a change but not the identification of the direction of this change (Micheyl et al., 2008) and is preferred over the more standard 2 AFC task in situations where subjects would have difficulty learning the basis for discriminating stimulus pairs (Kingdom and Prins, 2010). This would very likely be the case for discriminating positions/directions along arbitrary, oblique axes in 3D space.

**Figure 2:**
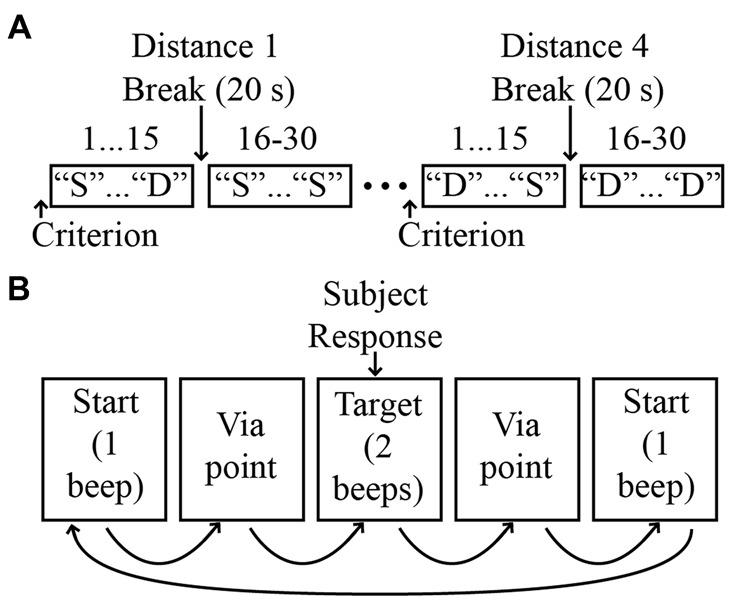
**(A)** Experimental protocol. Four distances were evaluated for each direction, with distance order randomized across directions. For simplicity, only two distances are shown. “S”: same; “D”: different. **(B)** Sequence of events on a single trial.

As shown in Figure 1C, our stimuli consisted of “judgment” positions that were located at different distances from the test position along a given direction. The spacing and orientation of the judgment positions could be easily manipulated in software, allowing for the testing of proprioception along any arbitrary direction/axis in 3D space. For a given movement direction, four judgment positions were used, which were spaced 1 cm apart in one of the six directions. As noted above, on a given trial, the two stimuli pairs could be the same or different. On “same” trials, the first and second stimuli were always the test position, while on different trials, the first stimulus was always the test position and the second was one of the other judgment positions. As illustrated in Figure 2A, each “different” stimulus was tested in a separate block, and the order of these blocks differed for different directions. For a given block of same-different trials, 30 trials were conducted (15 same and 15 different). These trials were performed in blocks of 15, separated by a short (~20 s) rest period. Within each 15-trial block, the same and different positions were randomized on a trial-by-trial basis. During the intervening rest periods, subjects were encouraged to view their arm, in order to minimize the possibility of proprioceptive drift (Wilson et al., 2010).

#### Experimental Protocol

Prior to performance of experimental trials, subjects performed 3–5 practice trials to ensure that they understood the task. Before each block of 30 trials subjects first experienced the robot moving their arm from the test position into the judgment position for that block in order to familiarize them with the testing environment and to ensure their comfort during the experiment. These “criterion” movements also provided subjects with information about the expected difference between positions in the given block. Subjects were instructed that on a given trial the robot would move their arm on a random path that would end either at the test position or the judgment position. Subjects were told that they should respond “same” if the judgment position was the same as the test position or “different” if it was not. If after 15 trials a subject still exhibited difficulty in understanding the task the subject was excused from the experiment and no further testing was conducted. If the subject had been performing the task but then informed the experimenters that they had been doing the task incorrectly the subject was also excused from the experiment and no further testing was conducted.

During blocks of experimental trials, subjects were instructed to remain as relaxed as possible and to avoid resisting or assisting motion of the robot. To minimize the possibility of muscular thixotropy on position sense (Proske and Gandevia, 2012), subjects were told to isometrically contract their arm muscles at the start of the experiment and before continuing after experimental breaks. This also served to reduce any fatigue or strain the subject was experiencing during the experiment. Figure 2B illustrates the sequence of events on a single trial. At the beginning of the trial the robot brought the arm to the test position and a single auditory tone was delivered. After a delay of 2 s the robot then moved the arm away from the test position to a random via point (maximum distance from the test position: 10 cm; minimum distance: 2 cm). After stopping very briefly (250 ms) at the via point, the robot then moved the arm either back to the test position (“same” trials) or to the judgment position (“different” trials) for that block. This was followed by the presentation of two auditory tones indicating the end of the trial. The subject was then required to respond “same” or “different”, indicating that the judgment position corresponded to either the original (test) position or the judgment position.

Movements to and from the via points were used to minimize the possibility that movement-related cues could be used to infer hand position (Wilson et al., 2010). These movements involved paths with a radius of curvature that was randomized between 2.44 cm and 15.8 cm (mean: 9.52). A linear velocity profile was used and the total movement time was fixed. As a result, peak velocities ranged from 1.2 cm/s to 7.8 cm/s (mean: 3.7 cm/s). As a result of these constraints, the total time between leaving the test position and arriving at any subsequent judgment position was also fixed at 3.75 s.

#### Data Analysis

Subjects’ responses were analyzed in MATLAB (The Mathworks Inc.). The proportion of trials where subjects responded “different” when the stimuli were different (*pH*), aka “hit rate”, and the proportion of trials where the subjects responded “different” when the stimuli were the same (*pF*), aka “false alarm rate”, were used to calculate *d’*, a measure of sensitivity derived from SDT (Kingdom and Prins, 2010). This measure is preferred over % correct (*Pc*) in most situations, as the latter can be greatly influenced by bias (i.e., a subject’s tendency toward “same” or “different” responses). *d’* was calculated as:

(1) $$d^{\prime }=z(pH)-z(pF)$$

where *z()* denotes a z-score transformation. For comparison we also report *Pc*, defined as:

(2) Pc=[pH+(1−pF)]/2

D’ was calculated for each distance and direction in both Experiments 1, 2. In Experiment 2 we also combined data for the leftward-rightward directions and forward-backward directions to facilitate comparison with previous results (Wilson et al., 2010).

##### Statistical Analyses on Population Data

All statistical analyses were conducted in SPSS 25. For Experiment 1, differences in d’ as a function of displacement distance and direction were assessed using a two-factor mixed analysis of variance (ANOVA; within subjects factor: distance; between subjects factor: direction). Multiple comparisons were conducted using Tukey’s HSD procedure.

Experiment 2 was designed to investigate the effects of arm posture on sensitivity. Analyses focused on displacement distances that showed the most variation across directions in Experiment 1 (i.e., 2 and 3 cm). For each distance, independent *t*-tests were used to compare sensitivity between arm postures for a given axis (forward/backward; leftward/rightward), as well as to compare sensitivity between axes for a given arm posture.

## Results

### Effects of Displacement Distance and Direction

**Figure 3:**
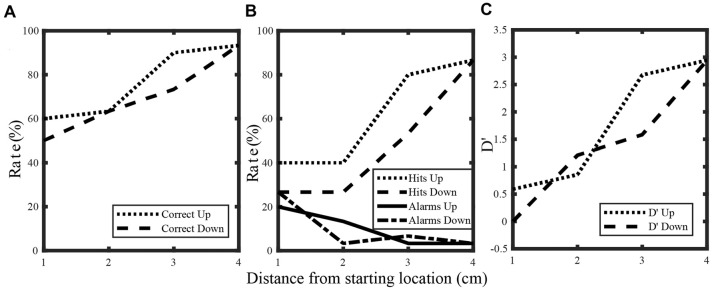
Percent correct **(A)**, hit rate and false alarm rate **(B)** and d’ **(C)** for a single subject. Data for the upward and downward directions are shown.

As expected, proprioceptive sensitivity increased monotonically with distance from the test position. Figure 3 shows plots of % correct (A), hit rates/false alarm rates (B), and d’ (C) as a function of distance from the starting (“test”) position. Data for the upward and downward directions are shown for a single subject. The plots for % correct show that for this subject, performance improved with distance and for the downward direction this increase was fairly linear. However, for the upward direction, trends with distance were somewhat different. Here, performance did not differ appreciably from 1 cm to 2 cm but improved rapidly from 2 cm to 3 cm. As a result of these differing trends, performance exceeded 75% correct (a standard threshold for discrimination) at 3 cm for the upward direction and 4 cm for the downward direction. Regardless of these differences, the overall trends with distance reflect the simple fact that discriminating between positions is more difficult when these positions are closer together than when they are spaced farther apart.

The plots for d’ and hit rate/false alarm rate highlight additional subtleties in this subject’s performance. Similar to % correct, d’ increased monotonically with distance for both directions. However, for d’ sensitivity at 2 cm differed somewhat between directions though no such differences were apparent for % correct. Differences between d’ and % correct can be understood from the hit rates and false alarm rates which are used to calculate d’. Even though the hit rate for the upward direction was greater than the hit rate for the downward direction at 2 cm, this subject also produced more false alarms for the upward direction. In other words, a bias towards “different” responses contributed strongly to the increased number of hits, rather than simply an increased ability to discriminate the positions effectively. As a result, sensitivity (as defined by d’) was actually somewhat larger for the downward direction. This shows that at the single subject level, d’ provides additional insights into discrimination than % correct alone can provide. As a result, analyses of sensitivity at the population level were focused exclusively on d’.

**Figure 4:**
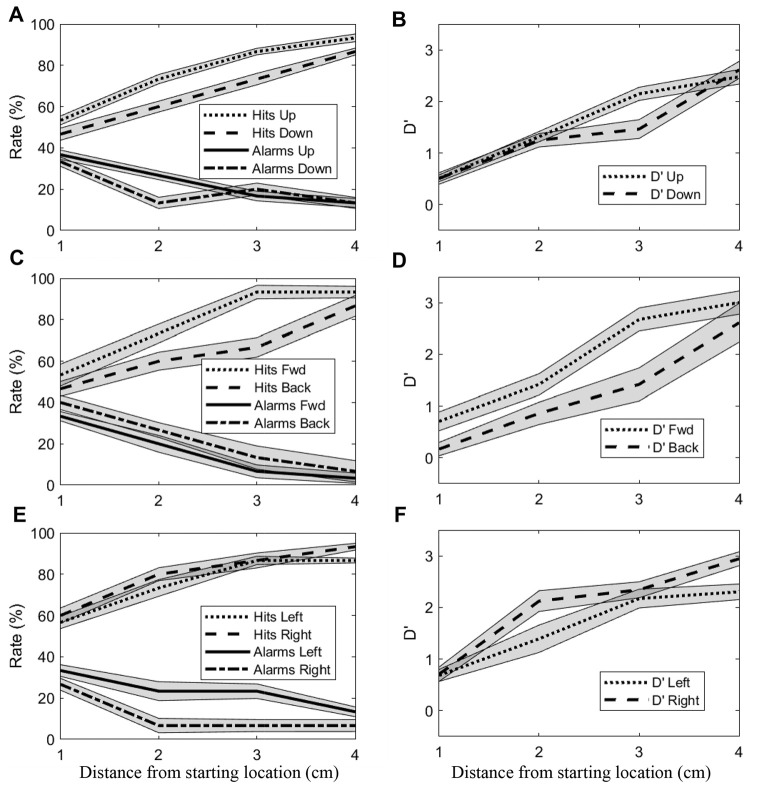
Mean (±SEM) hit rates, false alarm rates and d’ values for all subjects. **(A,B)** Upward and downward directions. **(C,D)** Forward and backward directions. **(E,F)** Rightward and leftward directions.

Effects of distance and direction were also evident at the population level. Figure 4 shows hit rates/false alarm rates and sensitivity (d’) as a function of distance for all directions. Data for opposing directions are shown separately in different rows. As was observed at the single subject level, d’ generally increased with distance for the upward/downward directions (Figure 4B). Greater sensitivity can be observed for the upward direction at 3 cm, with this difference appearing to arise from higher hit rates and somewhat lower false alarm rates for the upward direction. For the forward/backward directions (Figure 4D), sensitivity also increased with distance. Here, differences between directions were more consistent, with sensitivity for the forward direction appearing greater than for the backward direction at all distances up to 3 cm. Again, those differences appeared to be due both to higher hit rates and lower false alarm rates, in this case for the forward direction. For the leftward/rightward directions (Figure 4F), sensitivity only differed markedly at 2 cm, with rightward being greater than leftward. Here, the greater sensitivity did not appear to arise at all from higher hit rates, instead the observed differences were due almost entirely to substantially lower false alarm rates in the rightward direction.

**Figure 5:**
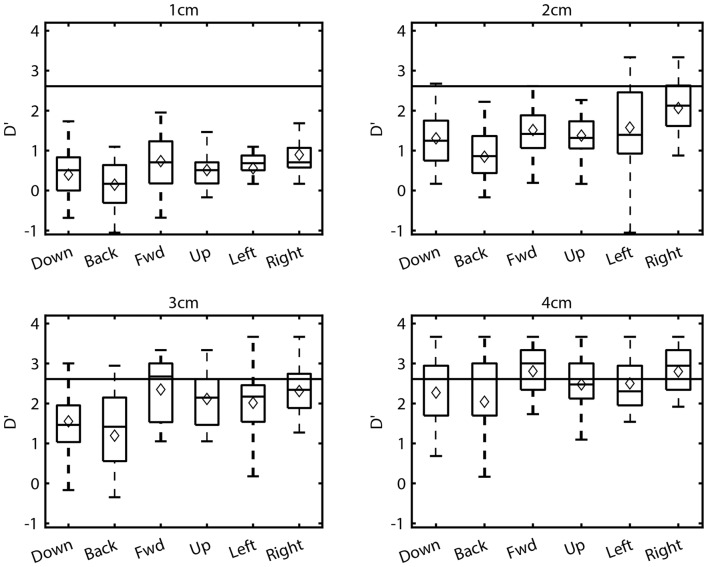
Boxplots of the sensitivities (d’) at each distance and direction for all subjects. Corresponding mean sensitivities (diamonds) are superimposed on each boxplot.

Although Figure 4 suggests that sensitivity was sometimes similar between opposing directions, isotropy was not a general finding. A two-factor ANOVA using data from all distances and directions revealed significant main effects of both factors on sensitivity (Distance: *F*_(3,360)_ = 348.04, *p* < 0.001; Direction: *F*_(5,120)_ = 6.36, *p < 0*.001), as well as a significant interaction effect (*F*_(15,360)_ = 2.60, *p < 0*.01). Such trends are clearly discernible in Figure 5. First, these boxplots illustrate that d’ values generally increased with distance for all directions. For example, for the downward direction mean d’ values for 1–4 cm were 0.4, 1.31, 1.55, and 2.27 respectively. Although d’ varied with distance for the other directions as well, trends with distance were not identical across directions. At 1 cm, d’ values were relatively low in magnitude and virtually identical for all directions while at 4 cm d’ was consistently larger but also comparable across directions. In contrast, clear differences in sensitivity across directions are apparent at the middle distances (2 and 3 cm). As a result, trends with distance were direction-dependent, as suggested by the ANOVA. To better illustrate this, we computed an estimate of the maximum sensitivity in this task by taking the global median across all directions at 4 cm (gray horizontal line). For the leftward and rightward directions, sensitivity approached this estimate of maximum sensitivity at a difference in position of 2 cm. This same level of performance was not reached until 3 cm for the upward and forward directions and not until 4 cm for the downward and backward directions. This is largely consistent with the *post hoc* Tukey tests, which showed that sensitivity for the downward (Mean = 1.29, SD = 0.98) and backward (Mean = 1.26, SD = 1.12) directions differed significantly from both the leftward (Mean = 1.83, SD = 0.94) and rightward (Mean = 2.05, SD = 0.95) directions (downward vs. leftward: *p* < 0.01; backward vs. leftward: *p* < 0.01; downward vs. rightward: *p* < 0.001; backward vs. rightward: *p* < 0.001). Thus, in this study the manner in which proprioceptive sensitivity improved with distance was anisotropic.

### Effects of Arm Configuration

Previous studies of arm proprioception in the horizontal plane have also reported direction-dependent differences in sensitivity. In particular, Wilson et al. (2010) demonstrated that proprioceptive acuity was greater for positions along a forward-backward axis than along a leftward-rightward axis. In contrast, in the present study, near maximal sensitivity was achieved at 2 cm for the leftward and rightward directions, with other directions (including forward and backward) reaching similar levels of performance only at 3 or 4 cm. This implies that in the present study, proprioceptive sensitivity was more acute for leftward/rightward directions than other directions, in apparent contradiction to the findings of Wilson et al. (2010). However, an important methodological difference existed between these two studies. In Wilson et al. (2010) the arm was contained within the same horizontal plane in which hand position was varied. In the present study the shoulder was adducted; therefore, the arm was rotated almost 90 out of the horizontal plane. To assess whether the apparent discrepancy between the two studies was due to the use of different initial arm configurations we conducted a follow-up experiment that assessed proprioceptive sensitivity along four directions using both adducted and abducted postures.

**Figure 6:**
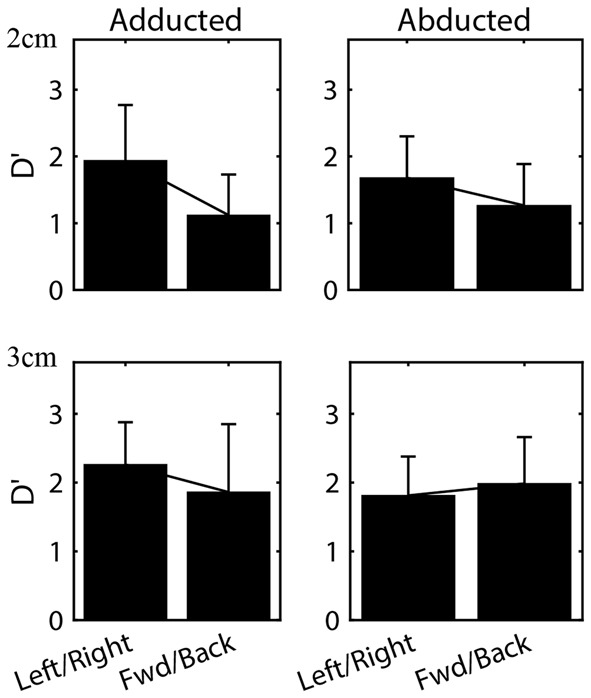
Mean (±SD) sensitivities for the leftward/rightward and forward/backward axes in both arm postures. Data for all subjects at the 2 and 3 cm distances are shown.

Varying initial arm posture resulted in changes in proprioceptive sensitivity. We first compared sensitivity between opposing directions for each arm posture. As in Experiment 1, no significant differences were found between the leftward and rightward directions or between the forward and backward directions for either arm posture. Therefore, to facilitate comparison with Wilson et al. (2010) data for opposing directions were grouped together for analysis. Figure 6 shows the mean (±SD) of the d’ values for each posture, grouped for the leftward/rightward and forward/backward directions. As expected given the results of Experiment 1, in the adducted posture sensitivity at 2 cm differed significantly between the leftward/rightward (Mean = 1.93, SD = 0.83) and forward/backward axes (Mean = 1.12, SD = 0.61; *t*-test, *t*_(75)_ = 4.86, *p < 0*.001). At 3 cm, sensitivity also differed significantly between axes in this posture (leftward/rightward Mean = 2.26, SD = 0.62; forward/backward Mean = 1.86, SD = 0.99; *t*_(79)_ = 2.20. *p < 0*.05). However, in the abducted posture no differences between axes were found (*p* = 0.07 and *p* = 0.4 for 2 and 3 cm, respectively). At least at 3 cm this lack of difference appeared to be due largely to a significant decrease in leftward/rightward sensitivity between adducted (Mean = 2.26, SD = 0.62) and abducted (Mean = 1.81, SD = 0.57) postures (*t*-test, *t*_(58)_ = 2.64, *p* < 0.05). In contrast, no statistically significant differences were found between postures for the forward/backward axis.

## Discussion

Several previous studies have characterized the proprioceptive abilities of human subjects within a horizontal plane. Among other findings, these studies have demonstrated that proprioceptive sensitivity varies with the direction of arm displacement as well as the position of the limb within the horizontal plane. Here, we employed a 7-DoF robot arm and analysis techniques derived from SDT to characterize proprioceptive abilities along several axes in 3D space, including opposing directions parallel to the gravity vector (i.e., upward and downward), which have not previously been characterized. Although our task involved comparing a currently felt position with a remembered one, previous studies have shown that performance on such tasks is similar to tasks without a substantial memory period (Wilson et al., 2010). Overall, we found that sensitivity depended on the distance between discriminated positions and, in agreement with previous findings, was also direction-dependent. The sensitivity profile for the upward direction was found to be similar to that of the forward direction and the profile for downward was similar to backward, with the latter two directions being the least sensitive overall at distances of 2–3 cm. In addition, when data were grouped together for opposing directions, sensitivity showed a dependence upon arm posture. These results suggest that arm proprioceptive sensitivity is both anisotropic and configuration-dependent in 3D space.

### Relevance to Clinical and Laboratory Assessment of Proprioception

Despite the importance of proprioception for normal perceptual and sensorimotor functioning, proprioception remains incompletely understood and its assessment in the clinic is primitive and limited in scope. One major factor contributing to the enigmatic nature of this “6th sense” is that there is no universally accepted method for assessing proprioception. In the clinic, assessment is performed in a relatively coarse manner and typically addresses only position sense. In one method, a patient’s joint (typically one of the digits of the foot or hand) is alternately moved in two opposing directions and the patient is asked to discriminate between these positions (e.g., “up or ‘down?”). A second commonly used method involves position matching, where the arm to be tested (‘test arm”) is passively moved into a test position and the subject is then asked to actively reproduce that position with either the same arm (after moving the arm back to its starting position) or with the contralateral arm. Although these tests can be quickly administered and are easy for patients to understand they are also suffer from several disadvantages. For example, such tests provide only coarse, discrete measures of proprioceptive abilities, i.e., proprioception is typically classified only as impaired or absent (Simo et al., 2014). Such tests are also currently thought to be associated with poor (or at least questionable) inter-rater (Lincoln et al., 1991) and/or intra-rater reliability (Lincoln et al., 1991; Carey, 1995; Dukelow et al., 2010; Simo et al., 2014). In addition, since these tests require physically guiding a subject through the required movement, proprioceptive estimates obtained this way can be contaminated by tactile, force and movement cues conveyed by the examiner (Simo et al., 2014). For these and other reasons, such methods are of limited usefulness in assessing proprioception outside of the clinical setting and were not employed in the present study.

Errors in reaching and pointing have been used in the laboratory to infer the contribution of proprioception to position sensing and movement control in both neurologically intact subjects (Flanders et al., 1992; Darling and Miller, 1993; Berkinblit et al., 1995; McIntyre et al., 1998; Vindras et al., 1998; Sober and Sabes, 2003; Goble and Brown, 2008; Apker et al., 2011) and patients (Blouin et al., 1993; Ghez et al., 1995; Gordon et al., 1995; Messier et al., 2003; Gosselin-Kessiby et al., 2009). Particularly relevant are studies involving movements without visual feedback of the moving hand (van Beers et al., 1998; Carrozzo et al., 1999; Apker et al., 2010; Apker and Buneo, 2012). Although these and other studies have provided a wealth of information about the relative roles of proprioception and vision in arm movement control, the active nature of reaching/pointing paradigms precludes isolation of the sensing aspect of proprioception from motor predictions derived from efference copy and an internal (forward) model. Although the contributions of sensory and motor processes can be partially disentangled using modeling and simulation techniques (Buneo et al., 1995; van Beers et al., 2004; Shi and Buneo, 2012), instrumented/robotic based assessment methods, which employ passive driving of the limb, can largely rule out the contribution of motor factors to proprioceptive function.

In the present study an instrumented (robotic) paradigm was used to quantify proprioceptive abilities. Early attempts at instrumented assessment typically allowed testing at only a single joint and still required the subject to actively move their limb or required the examiner to manually place the limb in position (Carey et al., 1996; Lee et al., 2003; Goble and Brown, 2008; Leibowitz et al., 2008). As a result of these limitations, several groups have recently employed the use of multijointed robots in proprioceptive assessment (Dukelow et al., 2010; Fuentes and Bastian, 2010; Wilson et al., 2010; Cressman and Henriques, 2011; Erickson and Karduna, 2012; Simo et al., 2014). Robotic assessment has several advantages over traditional manual assessments and other instrumented tests. Typically several joints can be assessed at once and can be done so relatively quickly (Dukelow et al., 2010). In addition, the limb does not have to be manipulated by the examiner which, as previously noted, can often provide subtle movement related cues to the subject. Second, the high spatial precision of modern robots means that errors in repeated positioning of the limb are nearly non-existent relative to manual positioning. Lastly, the ratio-level nature of the data that can be acquired using robots means that assessment can be more quantitative and more likely to reveal impairment (Dukelow et al., 2010; Simo et al., 2014). Thus, using a high precision device such as a robot can greatly improve the objectivity and reliability of proprioceptive assessments.

One potential limitation of the approach used here was that subjects’ arms were passively driven between positions. Since proprioceptors are most often stimulated during active movements, the extent to which our measurements provide a complete picture of proprioceptive abilities is unclear. However, recent work by Cressman and Henriques (2011) suggests that passive assessments may generalize well to at least some active contexts. These investigators assessed changes in perceived hand position after subjects either: (a) actively moved the handle of a manipulandum along a constrained linear path; or (b) had the handle passively moved along the same linear path (Cressman and Henriques, 2011). In both paradigms, once the hand reached the final position, subjects were required to make a 2 AFC judgment about the position of their hand relative to a visual reference marker. Following adaptation to altered visual feedback of the hand, proprioceptive estimates were found to be biased in the same direction as corresponding reaching movements, regardless of the nature of hand displacement (i.e., active or passive). This suggests that passive driving of the limb, as employed in the present study, may provide a robust estimate of proprioceptive abilities under a variety of contexts (i.e., active and/or placement displacement of the limb).

### Anisotropies in Position Estimation

Several previous studies have reported directional and workspace dependencies in proprioceptive abilities within the horizontal plane. For example, van Beers et al. (1998) studied the ability of human subjects to localize visual or proprioceptive targets at three positions in the horizontal plane. They found that subjects were more precise when localizing positions along an anterior-posterior axis than along an azimuthal one. In addition, subjects were more precise when localizing positions closer to the body than farther away. Subsequent work employing visuomotor adaptation paradigms (van Beers et al., 1999, 2002) confirmed the direction-dependent precision of both proprioceptive and visual localization and described some of the basic rules underlying the integration of information derived from these senses. The findings for proprioception were largely confirmed by a study involving direct examination of proprioceptive abilities employing a planar robot (Wilson et al., 2010). Here, subjects were required to judge the position of their hand with respect to either a remembered proprioceptive reference position or a visual reference. Judgment positions were attained via passive movements of the robots end effector (handle), which was grasped by the subjects. Proprioceptive acuity (i.e., sensitivity to change in hand position) was found to be greater for hand positions closer to the body and for changes in hand position occurring along an anterior-posterior axis. In addition, these investigators found limb-dependent differences in proprioceptive bias (perceived location of the hand) and also found that bias was reduced when the hand was closer to the body than farther away.

In previous studies, workspace and directional differences in proprioceptive abilities were explained by geometric factors. For example, identical changes in hand position performed at different locations in the workspace would be expected to result in different relative changes in joint angle, i.e., smaller changes in joint angle for positions further from the body and larger changes closer in (Wilson et al., 2010). As a result, muscle spindles would stretch to differing degrees at these locations, giving rise to the observed differences in proprioceptive abilities. A similar mechanism is thought to underlie directional differences in proprioceptive abilities. Although a geometric argument makes sense, in 2D experiments changes in limb geometry and changes in the position of the hand in the work space are naturally confounded. Thus, it’s unclear if the differences observed in 2D are entirely geometric in origin or if they arise in part from other factors, such as the frequency distribution of workspace positions naturally visited by the hand (Slijper et al., 2009) or asymmetries in the distribution of preferred sensory directions of arm muscle spindles (Bergenheim et al., 2000; Roll et al., 2000). In the present experiments however, hand position and arm configuration were not confounded. This lends support to the idea that geometric changes, including those that don’t alter the position of the hand in the workspace, are an important factor in determining arm proprioceptive sensitivity.

Regarding performance for directions outside the horizontal plane, neurophysiological studies have reported that fewer neurons in the rat dorsal spinocerebellar tract (Bosco and Poppele, 2001; Valle et al., 2007) and primate somatosensory cortex (Tillery et al., 1996) are tuned to movements/positions along the vertical axis than along other axes. This suggests that proprioceptive abilities should be diminished for arm displacements with substantial vertical components, a finding that was not generally observed here. That is, although sensitivity for the downward direction was poor relative to most other directions at intermediate distances, sensitivity for the upward direction was similar to the forward, leftward and rightward directions. The reasons for this discrepancy with neurophysiological studies are not immediately apparent. It should be noted however that some differences between the upward and downward directions were observed in this study. As mentioned previously, several studies have demonstrated robust differences in movement kinematics for upward vs. downward arm movements (Papaxanthis et al., 2003; Gentili et al., 2007; Le Seac’h and Mcintyre, 2007; Berret et al., 2008). Other work suggests these differences reflect the contribution of an internal model that is used in part to anticipate and exploit the anisotropic effects of gravity on the limb (Gaveau et al., 2016). This model, presumably acquired during development, would depend in part on the perceived effort associated with moving in different directions in the vertical plane, information which the proprioceptive system is ideally suited to provide (Proske and Gandevia, 2012). Thus, in addition to the aforementioned factors, anisotropic perception of limb position in 3D space could partially reflect the influence of an internal model that incorporates the perceived effort associated with moving in different directions relative to the gravitational vertical.

## Author Contributions

CB and JK conceived the experiments, analyzed data, prepared figures and drafted the manuscript. CB, JK, BW and PA designed the experiments, interpreted results of experiments, edited and revised the manuscript and approved the final version of manuscript. CB, JK and BW performed experiments.

## Conflict of Interest Statement

The authors declare that the research was conducted in the absence of any commercial or financial relationships that could be construed as a potential conflict of interest.
